# Genetic Polymorphisms of Superoxide Dismutase Locus of *Pneumocystis jirovecii* in Spanish Population

**DOI:** 10.3389/fpubh.2019.00292

**Published:** 2019-10-15

**Authors:** Rubén Morilla, Amaia González-Magaña, Vicente Friaza, Yaxsier de Armas, Francisco J. Medrano, Enrique J. Calderón, Carmen de la Horra

**Affiliations:** ^1^Department of Nursing, Universidad de Sevilla, Seville, Spain; ^2^Instituto de Biomedicina de Sevilla, Hospital Universitario Virgen del Rocío, Consejo Superior de Investigaciones Científicas, Universidad de Sevilla, Seville, Spain; ^3^Centro de Investigación Biomédica en Red de Epidemiología y Salud Pública (CIBERESP), Hospital Universitario Virgen del Rocío, Seville, Spain; ^4^Hospital Microbiology Department, Institute of Tropical Medicine “Pedro Kourí”, Havana, Cuba

**Keywords:** *Pneumocystis*, molecular epidemiology, superoxide dismutase, colonization, Spain

## Abstract

**Objective:** *Pneumocystis* pneumonia remains a major opportunistic infection in immunocompromised patients worldwide. Colonization with *Pneumocystis jirovecii* has recently gained attention as an important issue for understanding the complete cycle of human *Pneumocystis* infection. *P. jirovecii* Superoxide Dismutase (SOD) gene could be a molecular target with high clinical relevance, but the epidemiological information about SOD genotypes distribution is scarce. The aim of this work was to provide information about the prevalence of genotypes of *Pneumocystis* SOD among Spanish patients and to describe possible differences between colonized and *Pneumocystis* pneumonia patients.

**Methods:** we developed a cross-sectional study analyzing broncho-alveolar lavage fluid samples from 30 *Pneumocystis* pneumonia patients, 30 colonized patients, and 20 controls using a nested PCR protocol designed to amplify the sodA gene of *P. jirovecii*. The diagnostic yield of SOD Nested PCR was evaluated against the routine practice of mtLSUrRNA Nested PCR, which is considered the gold standard.

**Results:** SOD locus was amplified in 90% of *Pneumocystis* pneumonia patients, 10% of colonized patients, and none of controls. Genotype SOD1 was observed in 11 cases (52.4%) and genotype SOD2 in 10 cases (47.6%). Genotype SOD2 was observed only in *Pneumocystis* pneumonia patients while the genotype SOD1 was observed in both colonized and *Pneumocystis* pneumonia patients.

**Conclusions:** This study provides epidemiological information about SOD genotypes distribution in Spain, showing a low genetic diversity and a predominant presence of genotype SOD1 in colonized patients. SOP Nested PCR was more sensitive and accurate assay in *Pneumocystis* pneumonia patients than in colonized individuals.

## Introduction

*Pneumocystis* pneumonia (PcP) remains a major opportunistic infection in HIV-infected patients in both developed and developing countries and an emerging problem in immunocompromised patients without HIV infection worldwide (1). Today, the interest in *Pneumocystis* infection goes beyond PcP because a new spectrum of disease seems to emerge in immunocompetent individuals. The presence of *Pneumocystis jirovecii* in patients with underlying chronic diseases such as chronic obstructive pulmonary disease or interstitial lung diseases has been suggested to be a comorbidity factor (2, 3). But also, *Pneumocystis* colonization has gained attention as an important issue for understanding the complete cycle of human *Pneumocystis* infection.

For *Pneumocystis* infection diagnosis in humans, conventional or real-time PCR assays based on the amplification of the large subunit of mitochondrial ribosomal DNA (mtLSU rDNA) are the most commonly used, but many other sequences have been targeted. Among the most assessed sequences are the major surface glycoprotein, the small subunit of mitochondrial ribosomal DNA (mtSSU rDNA), the internal transcribed spacers, the thymidylate synthase, the dihydrofolate reductase, or the heat-shock protein (4).

Superoxide dismutases (SOD; EC 1.15.1.1) are ubiquitous key enzymes involved in the cellular defense against oxidative stress. These enzymes catalyse the first step of the detoxification of the superoxide anion in a metal cofactor dependent reaction and result essential in cellular protection against reactive oxygen species (ROS). There are several classed of SOD that have different cofactor metal present in the active site (5–7).

The gene encoding a manganese-dependent superoxide dismutase (MnSOD) has been characterized in *Pneumocystis* from rat, mouse, rabbit, pig, monkey, and human (8). Five genotypes have been described heretofore of which three are the most frequent: SOD1 (110C; 215T), SOD2 (110T; 215C), and SOD3 (110T, 215T) (9–11).

This gene has been used in a few epidemiological studies mainly into a multilocus approach (9–12) but the epidemiological information about SOD genotypes distribution is scarce.

The aim of this work was to provide information about the prevalence of genotypes of *Pneumocystis* SOD among Spanish patients and to describe possible differences between colonized and PcP patients.

## Materials and Methods

This study had a cross-sectional design and included 80 not selected successive male or female patients over age 18 years who were admitted to the bronchoscopy unit between 2006 and 2014 for evaluation of their disease at Virgen del Rocio University Hospital, of whom an bronchoalveolar lavage (BAL) specimen was available for analysis. Children were exclude of this study. In all cases, informed consent was obtained from the patients before obtain of BAL fluid. The study was approved by the hospital's ethics committee and it was performed according ethical principles regarding human experimentation contained in Declaration of Helsinki.

Thirty patients had AIDS-related PcP, 30 were Chronical Obstructive Pulmonary Disease (COPD) patients colonized by *P. jirovecii* without clinical or radiological signs of PcP and 20 were control patients with different lung diseases without *Pneumocystis* colonization or infection. All of them were Caucasian of Spanish origin. A single individual of control group was HIV-infected, other three were organs transplant recipients.

In all cases the presence or absence of *Pneumocystis* was confirmed by analyzing BAL samples with nested PCR amplification of the mtLSUrDNA gene of *Pneumocystis* (13). A colonized subject was defined as an individual without signs or symptoms of pneumonia from whom a respiratory sample was obtained that contained *Pneumocystis*-DNA detectable by PCR. A PcP patient was defined as an individual with a clinical pneumonia who had *P. jirovecii* in respiratory samples identified by microscopy or molecular methods without other causal agents.

The samples were separated into two aliquots of 250 μl that were cryopreserved at −20°C. DNA extraction was performed with commercial Kit NucleoSpin Tissue (Macherey-Nagel) following the manufacturer's recommendations. Extracted DNA was eluted with 55 μl of ultrapure water. Its concentration and purity (A260/A280) was determined, discarding A260/A280 values >2 and lower than 1.6, which would indicate contamination by proteins and RNA, respectively.

Samples that had been identified as positive by nested PCR of the mtLSUrRNA gene were further examined to study SOD gene. A nested PCR protocol was designed to amplify a locus of the *sodA* gene of *P. jirovecii* which included in the first amplification round, the use of the external primers MnSODFw(-5′-GGT TTA ATT AGT CTT TTA GGC AC-3′) y SODR4-(5′-CCA AGA ATA ACT TTG CCT TGA G-3′) to obtain a 584 bp fragment. The second round of amplification utilized the primers FS2-(5′-TCT TTC TCA TGA TTT GCT TGA GG-3′) and RS2-(5′-CTT TCC TAT ACC TAC CAC CAC C-3′) and yielded a 218 bp product. Rounds included 35 and 40 cycles of amplification, respectively. The PCR products were analyzed by electrophoresis on a 2% agarose gel containing ethidium bromide, and the bands were visualized by UV light. To prevent false positives due to contamination, pipette tips with filters were used at all stages. DNA extraction, preparation of the reaction mixture, PCR amplification, and detection were performed in different areas of the laboratory. In addition, a positive control was included in each reaction. To detect any cross-contamination, all PCR steps were performed with a negative control of sterile water. All experiments were repeated at least twice.

The products of positive nested PCR for SOD locus were purified by columns of Sephacryl S-400 (Amersham Pharmacia Biotech). Genetic characterization was performed by direct sequencing in the Genomics Service of the Institute of Biomedicine of Seville (IBiS), where they were injected into capillary sequencer ABI PRISM 3500 DNA Analyzer of Applied Biosystems.

The consensus SOD gene sequence was obtained from the NCBI web-page with the identification code and the polymorphisms were analyzed as previously described at position 110 (Y:C or T) and 215 (Y:C or T). Sequencing files were opened with Chromas lite 2.1.1 software and converted to FASTA format, in order to align the MEGA software vs. 6.0.

Finally, nested PCR for SOD assay was evaluated for sensitivity and specificity against the routine practice of Nested PCR for mtLSUrRNA gene, which is considered as gold standard. Specimens were considered true positive if they were positive by both PCR, and true negative if negative by both PCR. The diagnostic accuracy (ACC) was defined as follows: ACC = (TP + TN)/(P + N) in which TP = true positive; TN = true negative; P = number of positive and N = number of negative. In addition, we calculated the Youden's J statistic (also called Youden's index) as a single statistic that captures the performance of a dichotomous diagnostic test.

## Results

*Pneumocystis jirovecii* SOD gene was detected 30 out of 80 patients, in 90% (27/30) of PcP patients, in 10% (3/30) of COPD colonized patients and in none on control patients.

SOD typing was possible in 19 of the 27 PcP patients and in two of the three COPD patients in which SOD gene was detected by PCR. Genotype SOD1 (110C; 215T) was identified in 11 cases and genotype SOD2 (110T; 215C) was identified in 10 cases (Table 1).

**Table 1:** Characteristics of patients in study and results of the molecular typing by subgroups.

**Patients**	***N***	**Sex (% males)**	**Age** ($\bar{x}\pm \text{SD}$ **± SD, years)**	**Sequenced/ amplified samples**	**Genotype SOD1 (110C; 215T)**	**Genotype SOD2 (110T; 215C)**
PcP	30	*73.3%*	41.12 ± 12.05	19/27	9	10
COPD colonized	30	90.0%	66.19 ± 12.50	2/3	2	0
Non colonized	20	70.0%	47.21 ± 18.45	–	–	

*$\bar{x}$, Mean; sd, standard deviation; PcP, Pneumocystis pneumonia; COPD, Chronic obstructive pulmonary disease*.

SOD Nested-PCR results were compared with data of Nested PCR for mtLSUrRNA gene, regarded as diagnostic gold standard, and concordances calculated among the results obtained. There were SOD Nested-PCR agreement with mtLSUrRNA Nested-PCR in 90% of PcP cases, in 10% of COPD colonized patients, and 100% of non-infected controls.

The likelihood positive ratio, sensitivity, and specificity, Predictive Negative Value, Predictive Positive Value, diagnostic accuracy and Youden's index of SOD Nested-PCR in PcP and colonized COPD patients are showed in Table 2.

**Table 2:** Comparing characteristics and properties of SOD nested PCR as diagnostic test between PcP patients and colonized patients using mtLSU nested PCR as gold standard.

**Diagnostic variable**	**PcP patients**	**Colonized COPD patients**
Sensitivity, % (95% CI)	80% (62.7–90.5%)	10% (3.5–25.6%)
Specificity, % (95% CI)	100% (83.9–100%)	100% (83.9-100%)
PPV, % (95% CI)	100% (86.2–100%)	100% (43.8–100%)
NPV, %(95% CI)	76.92% (57.9–89%)	42.6% (29.5–56.7%)
FPR, %(95% CI)	0% (0–16.1%)	0% (0–16.1%)
FNR, %(95% CI)	20% (9.5–37.3%)	90% (74.4–96.5%)
Accuracy, %(95% CI)	88% (76.2–94.4%)	46% (33–59.6%)
Youden's index	0.8	0.1
Likelihood ratio (–), (95% CI)	0.2 (0.1–0.41)	0.9 (0.8–1.1)

*PPV, Positive predictive value; NPV, negative predictive value; FPR, False positive rate; FNR, False negative rate; PcP, Pneumocystis pneumonia; COPD, Chronic obstructive pulmonary disease*.

## Discussion

This study provides some epidemiological information about SOD genotypes distribution in Spain and shows that there are a low genetic diversity circulating in our area.

In our study, only two (SOD1 and SOD2) of the five genotypes described in the literature has been observed. These genotypes has been described as the most prevalent in several epidemiological studies (9, 10, 12, 14–16). Similarly, they are the only ones detected in other studies (12, 15). In this sense, the genotypic distribution of this locus in our region is similar to that described in London, UK, and Harare, Zimbabwe (15, 16) and differs from whose reported in France, Cuba and Portugal, where they have also detected other genotypes (9, 10, 14). These results support the possible existence of geographic differences in the distribution of these genotypes, but further of multicentre studies covering a larger sample would be needed for confirmation.

None of the genotypes seems to be predominant over the other among the PcP patients included in our study. Nonetheless, despite appearing in only two cases, it is noteworthy that the genotype found in the colonized subjects is the SOD1 (110C, 215T). This is consistent with data that suggest a potential association between this genotype with moderate or low microorganism load, which could be related with lower virulence and have clinical implications (11). However, it needs to be taken account that these are preliminary findings to be confirmed by future studies on a larger sample of patients.

In our study, differences between the values obtained for AIDS-related PcP and COPD colonized patients are probably due to different amplification rates obtained in each group due to lower parasite burden in colonized patients than in PcP patients (17).

The difficult to identify the SOD gene is due to it is a nuclear unicopy gene, while the mtLSU gene is located in mitochondrial ribosomes and therefore multicopy (4). This would explain why in colonized individuals where the parasite load is usually quite low, SOD gene is much more difficult to detect.

This characteristic could be useful in clinical use to distinguish colonized patients of PcP patients. However, SOP Nested PCR should not use alone for clinical diagnosis because, whilst it has a good specificity (100%), it has a low sensitivity (80%). Notwithstanding the foregoing, SOP Nested PCR could be used together with more sensitive PCR as those that target multicopies genes in a multiplex PCR as an early, discriminating, and accurate tool to diagnose PcP (18).

On the other hand, the lower sensitivity of SOP Nested PCR in colonized individuals (10%) and the poor diversity of genotypes limits its usefulness in multilocus genotyping studies that include not only PcP patients but also colonized individuals.

However, these conclusions need to be taken with a degree of caution as small size of study and because the information provided by a cross-sectional design is limited. Therefore, carrying out future more comprehensive studies to further define the role of SOP nested PCR in epidemiological studies of *Pneumocystis* infection and its utility in clinical diagnosis would be desirable.

## Data Availability Statement

The sequences of polymorphisms described in our paper can be found in Genbank under accession numbers: MG010739.1; MG010730.1.

## Ethics Statement

The studies involving human participants were reviewed and approved by Comité de ética de la investigación del Hospital Universitario Virgen del Rocío. The patients/participants provided their written informed consent to participate in this study.

## Author Contributions

EC and CH conceived and designed the research. AG-M and VF collected and analyzed the data. YA and FM performed the statistical analysis. VF, AG-M, CH, and FM contributed to the development of the study and interpreted the data. RM and EC wrote the draft of the manuscript. All authors reviewed and approved the final version of the manuscript.

### Conflict of Interest

The authors declare that the research was conducted in the absence of any commercial or financial relationships that could be construed as a potential conflict of interest.
